# A cost-analysis study of using adult red cell packs and Pedi-Packs in newborn intensive care units in Southern Iran

**DOI:** 10.1186/s12962-021-00267-7

**Published:** 2021-03-06

**Authors:** Sezaneh Haghpanah, Shima Miladi, Ali Zamani, Ali Mohammad Keshtvarz Hesam Abadi, Marjan Gholami, Maryam Gholami

**Affiliations:** 1 Hematology Research Center, Shiraz University of Medical Sciences, Shiraz, Iran; 2 Clinical Research Development Center, Nemazee Hospital, Shiraz University of Medical Sciences, Shiraz, Iran; 3 Blood Bank, Nemazee Hospital, Shiraz University of Medical Sciences, Shiraz, Iran

**Keywords:** Cost, Neonate, Newborns intensive care unit, Transfusion practice, Donor, Exposure

## Abstract

**Background and objective:**

Saving blood products is an important public health issue especially in developing countries with limited financial resources. We aimed to suggest a new hypothetical model to make a change in the current blood transfusion policy in the newborn intensive care unit (NICU) to reduce wastage of blood supplies as well as the risk of exposure to multiple donors.

**Methods:**

In this cross-sectional study, all transfused neonates (n = 70) who were admitted to NICU of Nemazee Hospital, a tertiary referral hospital in Southern Iran, were evaluated between March and June 2019. Based on the information of neonates’ transfusion during this study period and determined transfusion indices, a specific pediatric pack was suggested and the related total costs per transfusion, as well as the donor-exposure rate of the hypothetical and the current transfusion method, were compared.

**Results:**

Considering the mean number of transfusions per neonate: 4 and mean volume of transfused packed red cells: 20 ml per transfusion, the cost-analysis of pediatric and the adult pack was presented. Arithmetically, we proved a higher total cost per transfusion for using adult pack comparing to pediatric pack. Additionally, using a pediatric pack set leads to a 24% reduction in RBCs wastage per transfusion and a 68.13% reduction in donor-exposure rate.

**Conclusions:**

The assignment of a dedicated pediatric pack for neonates will be able to improve the cost-effectiveness by a substantial reduction in donor-exposure rate and blood wastage. This finding should be taken into consideration to generate economic growth and make improvements in child health status.

## Introduction

Despite carrying out some effective strategies such as micro methods blood sampling and autologous placental blood transfusion practice [1–3] to tackle blood loss in the Newborn intensive care unit (NICU), newborns are highly exposed to blood loss and anemia caused by repeated diagnostic phlebotomies [4, 5]. Therefore, multiple small-volume transfusions are often required to deal with this issue. Subsequently, exposure to multiple donors in red blood cells (RBCs) transfusion practice causes concerns about the risk of transmitted infections [6] as well as the hazard of respiratory distress syndrome, bronchopulmonary dysplasia, retinopathy of prematurity, and necrotizing enterocolitis [7, 8]. To reduce the exposure rate, developed countries have established some effective transfusion methods employing each blood donation for a particular newborn [7–16], such as using the programs with the technology of extending the RBCs stored up to 35–42 days by use of a sterile connection device. In this method, an appropriate volume is transferred by gravity into connected satellite packs whenever transfusion is required. [9, 14, 17–23].

On the other hand, using this program leads to reduce the rates of wastage of blood units compared to using RBCs stored up to < 5 or 7 days due to reserve and use of blood for more than one transfusion from the same donation unit [7, 15–17].

In the literature search, we did not find any document on the use of specific pediatric blood bags such as pediatric frozen red cell packs (Pedi-Packs) in Asian countries. However, the reported utilization rates reveal the lack of an optimal well-defined RBC transfusion method in this region as well [24–26]. Up to our knowledge, in Iran, we have no specific pediatric blood bag at present. Therefore, routine adult packs of fresh red cells (< 5 or 7 days of storage) are utilized for premature neonates and infants traditionally [27–29]. Consequently, due to small-blood volume consumption per transfusion, it not only results in multiple donors exposure but also causes wastage of a large number of blood supplies, as the proportion of 34.7% and 93% RBCs wastage have been reported in NICU and surgery units in different centers in Iran [30, 31].

Single donor program is a remarkable strategy to achieve a cost-effective method of neonatal transfusion, as a saving of $0.5 and $5.54 per transfusion were reported in two various studies, [18, 21]. Furthermore, assigning Pedi-Packs as a single donor program using at least 4 satellite packs for up to 35–42 days stored RBCs was reported as a safe, convenient, and effective method for transfusion in neonates in several other cost-effectiveness studies as well [9, 14, 17, 19, 20, 23].

Since we do not have any specific transfusion method for newborns and infants currently in Iran, we decided to notify the health policymakers not only in Iran but also in other developing countries with a similar strategy of this major health concern. Therefore, this study was designed to compare the donor exposure rates and the total cost of the hypothetical usage of Pedi-Packs with the routinely administered adult RBCs packs for neonates in NICU.

## Methods

### Subjects

In this cross-sectional study, medical charts of all neonates who were admitted to NICU of Nemazee Hospital, a tertiary referral hospital in Shiraz, Southern Iran were evaluated between March and June 2019. All newborn babies who received blood transfusion during 3 months of the study (n = 70) were considered as our study population. Based on the pilot study and estimating the wastage percentage (p = 92%), 34 cases were calculated as the least acceptable sample size for this study.

The study protocol was approved by the Ethical Committee of Shiraz University of Medical Sciences. (ID = IR.SUMS.REC.1398.995).

Blood Bank (BB) records of all participants were reviewed. Required information consisted of age, sex, weight, cause of admission, number of days stay in NICU, number of transfused packed red cell units (250 ml) and, the time interval between the first and the last transfusion for each neonate. Afterward, we calculated the mean number of transfusions and donor-exposure rate per neonate, as well as the mean volume of wasted RBCs per transfusion.

### Current transfusion practice

An adult RBCs pack of 250 ml fresh red cells stored with < 7 days, plasma reduced, CMV antibody-negative blood of the neonate’s ABO and Rh D group are requested and the cross-matching for each neonate requiring transfusion in the NICU are performed by BB. Small-volume blood is utilized for the first transfusion and if more transfusion is required after the expiry date of the first unit, a second blood unit will be used, and the remains of the first one is will be discarded.

### Hypothetical single donor exposure program

A Pedi-Pack set of fewer than 7 days old will be requested from the BB for each neonate requiring transfusion in the NICU. This set is leucocyte depleted (mean leucocyte count 2 × 10^6^/unit after filtration) which is prepared by the BB using a Sepacell R-500 filter in conjunction with a Sterile Connecting Device to preserve a 35-day shelf life for dedicating to one neonate. However, this duration can be extended to 42 days without adverse effects on pH, hematocrit, and potassium concentration of the stored blood [32, 33]. So, we can reserve it for each neonate up to our estimated time interval of transfusions as an expiry date [14, 23]. So, whenever transfusion is required, an appropriate blood volume from the labeled donation main unit is transferred into a satellite bag and supplied without further compatibility testing.

### Definition of blood transfusion costs

The total costs of the suggested Pedi-Pack and routine adult RBCs pack were compared arithmetically. For this purpose, we specified the total cost (TC) per transfusion for each of the two methods (Table 1) which consist of two components: acquisition RBCs costs and wasted resources cost per transfusion. The first component includes two subgroups: the first subgroup contained handling hospital blood bank's cost (HC) that is administrative activities associated with handling blood products as well as laboratory tests (LC) that both of them are performed by the hospital blood bank and are considered the same for both Pedi-Pack and adult pack; the second subgroup is defined as RBCs preparation cost (PC) which is different between the two methods as much as “x” and is related to blood the administration equipment used and the preparation process by the Blood Transfusion Organization (BTO). Moreover, the second component of the TC is the wasted resources cost consisting of the wasted RBCs cost (WC) that is the difference between the two methods as much as “f” and calculated by the wasted volume of packed RBCs multiplying in the final acquisitioned RBCs cost (HC + LC + PC) of one ml packed RBCs in each method. Another subgroup of the wasted resources is the transfusion risk cost (WRC) which is different between Pedi-Pack and adult pack as much as “m” (Table 1) [18, 34].

**Table 1:** Comparison of total costs of the suggested Pedi-Pack and routine adult RBC pack

		Handling hospital blood bank’s cost		HC			Constant for adult and pediatric unit
		Laboratory cost		LC			
Acquisitioned RBC cost (I)		RBC preparation cost by the BTO		PC		Adult pack	$PC_{a}$
						Pedi-Pack	[1] $PC_{p} = PC_{a} + x$^a^ $$\begin{gathered} PCp \, > \, PCa \hfill \\ [*1]_{{}} x < \, PCa^{{}} \hfill \\ \end{gathered}$$
Wasted resources cost per transfusion (II)		Wasted RBC cost per transfusion		$WC$		Adult pack	$WC_{a}$
						Pedi-Pack	$WC_{p}$
		Wasted RBC cost due to transfusion risk		$WRC$		Adult pack	$[2]_{{}} WRC_{a} = WRC_{p} + m$^b^
						Pedi-Pack	$WRC_{p}$
Total cost per transfusion = (I) + (II)		–		TC		Adult pack	$PC_{a} + WC{}_{a} + WRC_{a} + HC + LC$
						Pedi-Pack	$PC_{p} + WC_{p} + WRC_{p} + HC + LC$

^a^Estimated differential cost in preparation of Pedi-Packs vs. adult packs

^b^Estimated differential cost in wastage due to transfusion risk in adult pack vs Pedi-Pack

### Statistical analysis

Data analysis was performed using Statistical Package for the Social Sciences (SPSS) software version 18 (SPSS Inc., Chicago, IL, USA). Descriptive information was presented as mean, standard deviation, median, frequency, and percentage. Subsequently, a cost comparison between two methods considering determined transfusion indices was done arithmetically.

## Results

Demographic data and clinical characteristics of the neonates are presented in Table 2. The mean gestational age, birth weight of the neonates, and the number of days stay in NICU were 33.24 ± 2.52 (range 27–39) weeks, 2081 ± 680 (range 760–3800) gram, and 19.08 ± 18.04 (range 1–84) days, respectively, including 39 males (56%) and 31 (44%) females. Thirty-three (47%) had single-event (or multiple transfusions in one day) and 37 (53%) of the study population had multiple-event transfusions on different days. The most common clinical diagnosis on admission was congenital heart disease (8%), necrotizing enterocolitis (5.6%), and sepsis (2.8%).

**Table 2:** Demographic data and clinical characteristics of the study population

Variables		Mean ± sd (min–max)
Gestational age (week)		33.24 ± 2.52 (27–39)
Birth weight (gram)		2081 ± 680 (760–3800)
Number of days stay in NICU (day)		19.08 ± 18.04 (1–84)

		(%) N
Gender		
Male		(56) 39
Female		(44) 31
Clinical diagnosis		
Hyper bilirubinemia		(2) 5
Metabolic disorder		(1.2) 3
Congenital heart disease		(8) 20
Prematurity		(1.2) 3
Anemia		(2) 5
Necrotizing enterocolitis		(5.6) 14
Sepsis		(2.8) 7
Imperforated anus		(1.2) 3
Ileus		(0.4) 1
Respiratory distress syndrome		(1.2) 3
Nephrotic syndrome		(0.4) 1
Tracheoesophageal fistula		(0.8) 2
Developmental failure		(0.8) 2
Sacrococcygeal teratoma		(0.4) 1

The details of packed RBCs consumption and wastage using adult packs in NICU in the study period are described in Table 3. The total number of adult packs that were used in this duration was 221, while the total number of transfusions was 250, indicating that 29 units were shared amongst the neonates in this period. Moreover, the mean volume transfused packed red cells was 20 ml per transfusion and 71.42 ml per neonate during admission.

**Table 3:** Red cell consumption and wastage based on the routinely used transfusion practice during the study period

Variables	
Mean (median) duration between the first and the last transfusion (day)	20 (14)
Total number of the adult packs used	221
Total number of transfusions	250
Mean exposure to donor per neonate	221/70 = 3.16
Percent of reduction in exposure to donor per neonate	100 − (100(1 ×)/3.16) = 68.13%
Mean transfusion number per neonate	250/70 = 3.57
Number of satellite packs based on the mean transfusion number per neonate	4
Mean transfusion number per unit	250/221 = 1.11 ml
Total Volume of transfused RBCs	5000 ml
Mean volume of transfused RBCs per transfusion	5000 ml/250 = 20 ml
Mean volume of transfused RBCs per neonate	3.57 × 20 ml = 71.42 ml
Total volume of wasted RBCs	250 × 230 ml = 57,500 ml

Mean exposure to donor per neonate related to Pedi-Pack = 1

In our Pedi-Pack model, we suggest a total capacity of 250-ml packed red cells which are closed to the adult pack used, with 4 empty satellite packs also allowing up to 4 separate transfusions to be given based on the specified mean number of transfusion per neonate: 3.57 (nearly 4) during hospitalization in NICU. In the other words to simplicity in the further calculation, we can assume a pedi-pack set with 4 satellite packs each containing 62.5 ml RBCs (250 ml ÷ 4) constantly attached.

Also, since the mean volume of transfused RBCs per transfusion was calculated as 20 ml, 62.5-ml satellite packs can support the neonatal requirement per one transfusion.

Also, the expiration date of the Pedi-Pack set is considered at least 20 days based on the average time interval between the first and the last transfusion in the evaluated neonates.

Then, blood wastage based on using this model was calculated and compared with the volume of blood wastage using current transfusion practice in NICU (Table 4). The volume of wasted red cells per transfusion was determined 230 ml using adult pack compared to 42.5 ml using Pedi-Pack owning to mean volume of transfused RBCs per transfusion: 20 ml and considering that each sub-pack pack is used only once.

**Table 4:** Comparison of RBCs wastage between the routine (adult pack) and new suggested transfusion practice (Pedi-Packs) for neonates

Volume ml (%)		
Volume of wasted RBCs per transfusion^a^		
Adult pack 250 ml		250–20 = 230 ml (230 ml/250 ml = 92%)
Pediatric pack 250 ml		62.5–20 = 42.5 ml (42.5 ml/62.5 ml = 68%)
volume of wasted RBCs per neonate^a^		
Adult pack 250 ml		4 × 230 ml = 920 ml
Pediatric pack 250 ml		4 × 42.5 ml = 170 ml

^a^Regarded to average volume of transfusion per unit (= 20) and the number of satellite packs (= 4)

In the next step, we compared the total cost per transfusion between the two methods (Table 1).

As mentioned above, components of HC and LC are equal in both methods, so the differences between the total costs of Pedi-Pack and adult pack arise from variations in PC, WC, and WRC.

It seems that PC in Pedi-Pack is more than an adult pack that we considered this difference as much as “x”, Eq. (1) ($^{{}} PC_{p} = PC_{a} + x$), [*1] ($x < \, PCa$). The estimated values of the $WC_{a}$ $WC_{p}$ and are calculated as demonstrated in Eq. (2) which resulted in the Eq. (3) ($\mathop {\mathop {WC_{a} = }\limits_{{}} }\limits_{{}} WC_{p} + f$) whereas the “f” value was specified as the differential cost in the wasted RBCs. Afterward using Eqs. (1, 3), and (4) ($WRC_{a} = WRC_{p} + m$), we were able to prove that the $TC_{a}$ can be higher than the $TC_{p}$, if and only if the burden cost of purchasing dependent on Pedi-Pack (x) is lower than the total burden costs of wasted resources related to adult pack (f + m) (Eq. (6).

1 $$\it \left[ \begin{gathered} RBCs \, Cost \, per \, ml = (Acquisitioned \, RBC \, cost)/250 = (PC + HC + LC)/250 \hfill \\ \hfill \\ WC_{{}} per_{{}} transfusion = Wasted \, RBCs \, Volume^{{}} per _{{}} transfusion_{{}} *^{{}} RBCs \, Cost \, per \, ml \hfill \\ \end{gathered} \right].$$

2 $$\buildrel {HC, LC\, are\, constant\, so\, are\,removed\, from\, both\, arms} \over \longrightarrow \left[ \begin{gathered} WC_{a} = 230 * \frac{{PC_{a} }}{250} = 0.92_{{}} PC_{a} \hfill \\ WC_{p} = 42.5 * \frac{{PC_{p} }}{250} = 0.17_{{}} PC_{p}\end{gathered} \right].$$

$$\begin{gathered}{\rm we\,need\,to\,prove\,that}\, \langle0.92PC{_{a}} > 0.17PC_{p}\rangle\odot \hfill \\ If\, PC_{p} = PC_{a} + x [1] so 0.17PC_{p} = 0.17(PC_{a} + x ) \to 0.17PC_{p} = 0.17PC_{a} + 0.17x \hfill \\ Also\, since \left\{ {\begin{array}{*{20}{l}} {x > 0}\\ {P{C_a} > 0}\\ {x < P{C_a}[*1]} \end{array}} \right\} thus\, \forall b > 0 \quad x < b PC_{a} \hfill \\ So\,as\,{\frac{0.92 - 0.17}{0.17} > 0} \to x < ((0.92 - 0.17)/0.17)PC_{a} \hfill \\ finally\, 0.92 PC_{a} > 0.17 PC_{a} + 0.17x{\longrightarrow}0.92PC_{a} > 0.17 (PC_{a} + x) \hfill \\ {\longrightarrow} \langle 0.92 PC_{a} > 0.17PC_{p} \rangle \odot \hfill \end{gathered},$$

3 $${\longrightarrow}WC_{a} > WC_{p} \,\,{or\,on\,the\,other\,words}\,\, WC_{a} = WC_{p} + f.$$

4 $$Additionaly,_{{}} WRC_{a} = WRC_{p} + m.$$

5 $$\left[ \begin{gathered} TC_{a} = PC_{a} \, + WC_{a} \, + WRC_{a} \mathop {}\limits_{{}}^{{}} \hfill \\ TC_{p} = PC_{p} \, + WC_{p} \, + WRC_{p} \mathop{\longrightarrow}\limits^{[1],[3],[4]}TC_{p} = PC_{a} \, + WC_{a} \, + WRC_{a} + \underbrace {x - (f + m)}_{{}} \hfill \\ \end{gathered} \right]_{{}}.$$

6 $$Therefore,if_{{}} \left\{ \begin{gathered} x > (f + m)_{{}} \mathop{\longrightarrow}\limits^{{}}TC_{a} < TC_{p} \hfill \\ x < (f + m)_{{}} \mathop{\longrightarrow}\limits^{{}}TC_{a} > TC_{p} \hfill \\ \end{gathered} \right.$$

This wasted cost was calculated for each transfusion. It is noteworthy to consider that each newborn baby underwent 4 transfusions on average during their hospital admission, therefore the cost of wasted red cells per neonate is estimated as 4 times more using adult pack versus Pedi-Pack per neonate.

Moreover, looking at Table 4, if the wasted RBCs of the adult pack to Pedi-Pack is estimated 92–68% per transfusion, it can be explained that the Pedi-Packs have 24% less wastage than the adult packs.

On the other hand, the mean number of donor-exposure rates in the adult pack system was 3.16 compared to 1 in Pedi-Pack that is equivalent to a 68.13% reduction in donor-exposure rate using Pedi-Pack.

## Discussion

The total cost of the routine adult pack and the suggested Pedi-Pack was compared based on the assessment of packed RBCs utilization in three months in NICU. We proved that using Pedi-Pack instead of the routine adult pack with < 7 days of storage in NICU can decrease the total cost of transfusion and donor-exposure rate in neonates.

The volume of our hypothetical quadruple Pedi-Pack set was similar to what was reported by Satyam et al. and Cook et al. studies [9, 10]. However, other studies reported different values [11, 12, 15]. The mean volume of transfused RBCs per infusion in our study was determined 20 ml that was similar to the result of Kirsten et al. study [11].

We demonstrated that usage of the quadruple Pedi-Packs per neonate with a 20-day expiration date can decrease the donor-exposure rate from 3.16 to 1, which shows a 68.13% reduction in exposure (Kerstin et al., Wood et al., Cook et al., Ibojie et al., and Straaten et al. had 33%, 30%, 15%, 35.1%, 22% reduction in DE, respectively) [10, 11, 14, 16, 19]. Accordingly, several studies succeeded to prove a reduction in donor-exposure rate by replacement of the alternative blood transfusion methods in NICU using diverse donor programs such as the “sterile docking device” [18, 21], or pack-sets with 4–8 satellite packs, by dedicating single donor units for just single neonate with increasing the expiration date of RBCs pack [14, 19, 22, 23].

Based on our results, using Pedi-Pack sets can also be associated with 24% fewer RBCs wastage compared to adult packs. It is consistent with some other studies that show using the limited donor program followed by the specific pack sets such as Pedi-Packs may contain an additional cost, but global costs decrease due to reduction in the wasted resources associated costs of risk of exposure to multiple donors and the RBCs wastage which consequently increases the cost-effectiveness [18, 21]. Hilsenrath et al. reported a 44% reduction in donor-exposure rate, and despite the higher RBCs preparation cost, they could save $0.5 per transfusion leading to an increase the cost-effectiveness [18]. Similarly, Kakaya et al. calculated $35 for Pedi-Pack cost compared to $26 for the one-time transfusion cost [20]. They concluded that despite the higher cost, the Pedi-Pack can be more economical because it uses for more than one transfusion. Moreover, it has less risk of exposure to the donor for infants.

Our study was limited because our cost analysis was based on the hypothetically model rather than actual usage due to the unavailability of the pediatric pack and low financial support for intentional production of this specific type of blood bag. For instance, in the calculation of the total wasted resources which consisted of wasted RBCs cost and wasted costs related to clinical complications or WRC, we were able to compare only the first one, but due to the lack of Pedi-Packs set, we were not able to compare clinical complications΄ cost between the two methods in reality. However, this issue was shown previously that reducing the number of exposure to donor leads to a significant impact on reducing the clinical complications [18]. So as you noticed in mathematical terms we were not able to calculate the exact specified differential cost of RBCs preparation (x) in the Pedi-Pack set and the summation of burden costs related to donor exposure risk (m) and wasted RBCs (f), and thus, it was not possible to calculate the exact total costs. However, according to the reduction in wasted RBCs with Pedi-Pack (24% per transfusion) compared to adult pack as well as the higher rate of clinical complications due to higher number of exposure to the donor in the adult pack compared to Pedi-Pack (3.16 times) the value of "x" is not expected to exceed “f + m”, because the related costs of donor-exposure complications are generally higher than the RBCs preparation cost, so the application of new method would seem to be more cost-effective with so high possibility.

Taken together, our hypothetical model made us be able to show increased cost-effectiveness using Pedi-Packs compared to the routine adult pack for new borne babies admitted in NICU. Our results are in line with the implementation of alternative programs in practice leading to impressive outcomes in previous studies [17, 18, 21, 22]. The results of this study can be very helpful for policymakers especially in developing countries with a shortage of financial resources.

The next step recommended based on the result of this study, is providing the suggested Pedi-Pack by a collaboration of BTO and medical equipment companies to investigating this method in practice and establishing it as a cost-efficient method of pediatric transfusion.

## Conclusion

A simple change in policy with dedicating the pediatric pack set with preserving RBCs for up to its expiry date for newborns during their hospitalization will be able to considerably reduce donor exposure rate which poses severe threats to this high-risk group Moreover, the establishment of such an effective strategy will result in keep RBCs resources, which in turn is economically important particularly in developing countries with a shortage in financial resources.

## Data Availability

The methodology is listed in detail in the Methods section of the manuscript. All data are available upon request.

## Abbreviations

RBCs: Red blood cells
BB: Blood bank
NICU: Newborn intensive care unit
Pedi-Packs: Pediatric packs
TC: Total cost
HC: Handling hospital blood bank’s cost
LC: Laboratory tests cost
PC: Preparation cost
BTO: Blood transfusion organization
WC: Wasted RBCs cost
WRC: Wasted resources cost
SPSS: Statistical package for the social sciences
